# Hmgcs2 regulates M2 polarization of macrophages to repair myocardial injury induced by sepsis

**DOI:** 10.18632/aging.204944

**Published:** 2023-08-09

**Authors:** Xiao-Zheng Zou, Jun-Feng Hao, Ming-Xiao Hou

**Affiliations:** 1 Department of Critical Care Medicine, The Fourth Affiliated Hospital of China Medical University, Shenyang 110032, Liaoning, PR China; 2 Department of Cardiovascular Surgery, General Hospital of Northern Theater Command of China Medical University, Laboratory of Rescue Center of Severe Wound and Trauma PLA, Shenyang 110016, Liaoning, PR China; 3 Department of Nephrology, and Guangdong Provincial Key Laboratory of Autophagy and Major Chronic Non-communicable Diseases, Affiliated Hospital of Guangdong Medical University, Zhanjiang 524001, Guangdong, PR China; 4 Shenyang Medical College, Shenyang 110034, Liaoning, PR China; 5 Department of Cardiovascular Surgery, The Second Affiliated Hospital of Shenyang Medical College, The Veterans General Hospital of Liaoning, Shenyang 110001, Liaoning, PR China

**Keywords:** sepsis, CLP, Hmgcs2, M2

## Abstract

The respiratory and cardiovascular systems are often the most severely impacted by the rapid onset of sepsis, which can lead to multiple organ failure. The mortality has ranged from 10 to 40% when it has evolved into septic shock. This study sought to demonstrate the potential and role of Hmgcs2 in safeguarding against cardiovascular harm in septic mouse models.

The cecal ligament and puncture (CLP) model was used to induce sepsis in C57BL/6 mice, with Hmgcs2 expression in the myocardium of the mice being heightened and inflammatory factors being augmented. Subsequently, we utilized ASOs to silence the hmgcs2 gene, and found that silencing accelerated septic myocardial injury and cardiac dysfunction in CLP mice models. In contrast, hmgcs2 attenuated inflammation and apoptosis and protected against septic cardiomyopathy in murine septicemia models. Src production, spurred on by Hmgcs2, triggered the PI3K/Akt pathway and augmented M2 macrophage polarization. Moreover, the inhibition of M2 polarization by an Src antagonist significantly contributed to apoptosis of cardiomyocytes.

Our research revealed that Hmgcs2 inhibited the activation of pro-inflammatory macrophages and, through Src-dependent activation of PI3K/Akt pathway, promoted the anti-inflammatory phenotype, thus safeguarding myocardial damage from sepsis. This offers a novel theoretical basis for prevention and treatment of infectious complications.

## INTRODUCTION

The life-threatening condition of sepsis, a result of an abnormal immune reaction to infection, can cause organ dysfunction [1]. Cardiac dysfunction, a complication of sepsis, is a common occurrence, with around 60% of septic patients being diagnosed within the initial 3 days [2]. This complication is multifaceted, involving inflammatory reactions, oxidative stress damage, mitochondrial dysfunction, and apoptosis [3, 4]. Targeting regulation of macrophage polarization may be a beneficial way to restore the host immune balance, thus offering a novel therapeutic approach for sepsis treatment. Numerous experiments have demonstrated that cardiac dysfunction is connected to changes in cardiac substrate and energy metabolism. Ketone bodies have been observed to provoke oxidative stress and upregulate several signaling pathways linked to diabetic issues [5]. The metabolic and bioenergetic pathways point to ketone body metabolism as a significant factor in the myopathic heart [6]. Mitochondrial 3-hydroxy-3-methylglutaryl-CoA synthase 2 (Hmgcs2), part of the HMG CoA family of proteins, is the enzyme responsible for the rate-limiting reaction of ketogenesis [7]. This process takes place mainly in the liver mitochondrial matrix, with a rate that is proportional to total fat oxidation. Transported through the mitochondrial membrane via the acyl chain and β-oxidation, the mitochondrial isoform of Hmgcs2 catalyzes the condensation of acetoacetyl-COA (AcAc-COA) and acetyl-CoA (acetyl-CoA), resulting in the production of HMG CoA. HMGCL then cleaves HMG CoA, releasing acetyl-CoA and acetoacetate (AcAc), which is then taken up by phosphatidylcholine-dependent mitochondrial d-β OHB dehydrogenase (BDH1) and reduced to d-β-Hydroxybutyrate (d-βOHB) [7]. The regulation of Hmgcs2 expression and activity during early postnatal life, aging, diabetes, starvation, and the ingestion of a ketogenic diet [8] is still not fully understood, and its physiological status is still being explored.

The immune system’s dysfunction is the foundation of sepsis, a systemic inflammatory response syndrome brought on by infection. Macrophages, a key component of the innate immune system, are highly diverse and can be programmed to act in certain ways. The process of macrophage polarization involves a complex regulatory network, which is influenced by multiple signaling molecules, transcription factors, epigenetic modifications, and metabolic reprogramming [9]. M1-like macrophages release many pro-inflammatory mediators, whereas M2-like macrophages release many anti-inflammatory mediators. The disparity between M1-like and M2-like macrophages sparks the commencement and growth of sepsis [10–14]. Therefore, targeted regulation of the macrophage polarization process may be a useful approach to normalizing the host immune balance, providing a new therapeutic modality for the treatment of sepsis.

## MATERIALS AND METHODS

### Animals experiment

The sepsis model was induced via the CLP method. A selection of 24 male mice, each with an age of 6 weeks and a weight ranging from 22-25 g, was made using the Ethical Lot Number CMU2021201. All mice were randomly divided into (1) Sham group (*n* = 6): Equal dose control buffer was injected intraperitoneally every two weeks, and PBS (0.01 mol/L, pH7.4) was used as a control buffer; (2) Sham+ ASO group (*n* = 6): Mouse Hmgcs2 targeted antisense oligonucleotide (ASO, ISIS 191229;5′-CTGTTTGTCACTGTGGATG) was injected intraperitoneally every two weeks (25 mg/kg);(3) CLP group (*n* = 6): The same dose of PBS was injected intraperitoneally every two weeks;(4) CLP+ASO (*n* = 6): Mouse ASO (25 mg/kg) was injected intraperitoneally every two weeks. CLP modeling of the mice was performed at the 10th week of age. Following the induction of sepsis, mice were anesthetized with 100 mg/kg ketamine (HUONS, Seoul, Korea) and 10 mg/kg xylazine (Bayer Korea, Seoul, Korea) for 24 hours. After performance of a cardiac ultrasound, orbital blood was drawn and the heart was harvested for Western blot analysis and histological examination. Blood samples were anticoagulated with 10% sodium ethylenediamine tetraacetate (EDTA) at 4°C for 3 hours, centrifuged at 3000 rpm for 10 minutes, followed by serum collection which was kept in a refrigerator at a temperature of −80°C.

In strict compliance with the National Organizations of the Guide for the Care and Use of Laboratory Animals of Health, the study was conducted, and all operations on mice were sanctioned by the China Medical University Standards for the Laboratory Animals Welfare and Ethical Review (CMU 20211201) with a permit.

### Echocardiography

The Vevo 2100 system (Visual Sonics) was employed to assess cardiac contractile function through transthoracic M-mode echocardiography.

### Histopathological analysis

Hematoxylin and eosin (H&E) staining from Beijing Solabao, China (G1120) was used to assess the histochemistry of the remodeled animals. After 10% paraformaldehyde buffer had been used to fix mouse heart tissues for 24 hours, gradient alcohol dehydration and paraffin coating were performed. Subsequently, 5 μm thick paraffin sections were cut and dried in a 37°C incubator. Subsequently, xylene dewaxing was performed, and the slides were then subjected to H&E staining kits in accordance with the manufacturer’s instructions.

### Tissue immunofluorescence

Fluorescein-labeled antibodies were used against the respective antigens. PBS was replaced every 10 min to maintain the specimen humidity. The specimen was covered with fluorescent-labeled antibodies and stored in an enamel box for 30 min. The glass slide was rinsed with PBS solution in three cylinders, each washed for 3–5 min under oscillation. The glass slide was observed under a fluorescence microscope, after which a drop of glycerol was added to cover it, and the surplus moisture removed. The primary antibodies CD163 (1:500, Abcam, ab182422) and Arg1 (1:500, Abcam, ab96183) came from Abcam (Cambridge, London, UK), and the secondary fluorescence immunoglobulin G came from Abmart (1:1000, M21014S, Shanghai, China).

### Serum myocardial injury indicators

Using the Elabscience enzyme-linked immunosorbent assay (ELISA) kit (Wuhan, China), 24 hours post-reperfusion, the concentrations of creatine kinase MB isoenzyme (CK-MB, E-EL-M1203c), lactate dehydrogenase (LDH, E-EL-M0419c), and troponin I (cTnI, E-EL-M1203c) in the serum were determined in accordance with the instructions.

### Cell culture

From the American Type Culture Collection (ATCC, Manassas, VA, USA), THP-1 cells were procured and cultured in RPMI-1640 medium (high quality 10% fetal bovine serum (FBS); 0.05 mM β-mercaptoethanol; 1% penicillin/streptomycin). Subsequently, 100 ng/ml phorbol myristic acid acetate (PMA) was administered to THP-1 monocytes to induce M0 macrophages for 24 hours. Subsequently, M1 macrophages were exposed to 50 ng/ml LPS for 48 hours, while M2 macrophages were incubated with 20 ng/ml human IL-4 for the same duration. M1-like macrophages are the product of the iNOS pathway, which produces citrulline and NO from arginine, while M2-like macrophages are the product of the arginase pathway, which produce ornithine and urea [15] from arginine. CD86 and CD80 are the known M1 markers, and CD163 and CD206 are the known M2 markers [16]. Characterized by proinflammatory and antimicrobial effects, M1 macrophages release copious amounts of proinflammatory factors, such as IL-6, IL-18, IFN-γ, and TNF-α, whereas M2 macrophages are distinguished by anti-inflammatory effects, such as IL-10 [17]. Employing ELISA and qRT-PCR, we were able to detect the inflammatory factors IL-6, IL-1β, TNF-α, and IL-10. Additionally, flow cytometry was utilized to detect the surface markers (PE anti-human CD86 and APC anti-human CD206, both from BioLegend, San Diego, CA, USA) of stimulated cells.

Culturing AC16 human cardiomyocyte lines in DMEM: F12, supplemented with 10% FBS, 1% penicillin-streptomycin, and 1% fungizone (Invitrogen), was done in a humid atmosphere of 5% CO_2_ at 37°C until reaching a confluence of 70–80%.

### Cell transfection

The over-expressed Hmgcs2 lentivirus was conceived and synthesized by GeneChem (Shanghai, China). Full-length cDNA encoding Hmgcs2 was amplified from pCMV3-Hmgcs2 plasmid then inserted into pCDH-CMV-MCS-EF1-Puro vector to obtain Hmgcs2-overexpressing plasmid pCDH-Hmgcs2. HEK293T cells were co-transfected with pCDH-Hmgcs2 and packaging plasmids (pMD2 and pAX2) to obtain lentivirus particles of pCDH-Hmgcs2. Following this, the cells were transfected with the relevant reagents, following the manufacturer’s instructions, using Lipofectamine™ 3000 (Thermo Fisher Scientific, Waltham, MA, USA). THP-1 cells were infected with concentrated pCDH-Hmgcs2 lentivirus particles and then screened by puromycin to obtain stable Hmgcs2-overexpressed THP-1 cell line. Subsequent experiments employed the Src selective inhibitor PP1(1 μM), with cells pre-treated for 1 h.

### Cell counting Kit-8 assay (CCK-8)

AC16 cells were co-cultured with different condition mediums of macrophages for 24 h; AC16 (2 × 10^3^) were seeded into 96-well plates (Corning, NY, USA). At the designated hour, 10 μl of CCK-8 (Beyotime, Jiangsu, China) was added to each well. After 1 hour of incubation at 37°C, the absorbance at 450 nm was determined using an automatic microplate reader (Synergy4; BioTek, Winooski, VT, USA).

### Co-culturing and conditioning of the medium preparation with macrophages

The AC16 cells were washed and cultured with fresh medium or CM from macrophages with or without overexpressed Hmgcs2 lentivirus or PP1, to assess the effects of macrophages regulated by Hmgcs2/Src on AC16 cells. To obtain a conditioned medium (CM) of macrophages, the cell medium was collected and centrifuged at 3000 rpm for 10 minutes at 4°C.

### Western blot (WB)

Tissue proteins were extracted according to kit instructions (Sigma Aldrich, R0278, St. Louis, MO, USA). Quantifying protein concentrations and samples was done routinely with a BCA kit (Beyotime Biotechnology, P0010S, Beijing, China) from Louis, MO, USA. SDS-PAGE of 30 μg was conducted on each sample using either 8% or 12% SDS-PAGE, and then the semi-dry transfer was made to PVDF membranes. After blocking with 5% skim milk prepared with TBST for 1 h at room temperature, the membranes were incubated overnight at 4°C with pre-diluted primary antibodies. The secondary antibody was then incubated with the membrane for 1 hour at room temperature after it had been washed. The dilution ratios of all antibodies were as follows: Hmgcs2 (1:500, Abcam, ab137043, Cambridge, London, UK), Src (1:500, Abcam, ab109381, Cambridge, London, UK), p-PI3K (1:500, CST, Cell Signaling Technology, #17366, Danvers, MA, USA), PI3K (1:500, CST, #4292), p-AKT (1:500, CST, #4060), AKT (1:500, CST, #4685), and GAPDH (1:500, CST, #97166).

### Real-time quantitative PCR analysis (RT-qPCR)

Trizol and enzyme RNA Extraction Kit (Takara, RR820A, China) were employed to extract total RNA from the heart, and 1 μg of the RNA was reverse-transcribed with a reverse transcription Kit (Takara, RR047A, China). Real-time quantitative PCR was then conducted with gene-specific primers and a 7500abi biological system machine, and the absolute mRNA number was determined by comparing it to the threshold.

### Enzyme-linked immunosorbent assay (ELISA)

Using ELISA kits from Elabscience (Wuhan, China), the concentrations of IFN-β (E-MSEL-M0003), TNF-α (E-EL-M3063), IL-6 (E-EL-M0044c), IL-1β (E-MSEL-M0003), IL-10 (E-EL-M0046c) in the mice serum and TNF-α (E-EL-H0109c), IFN-β (E-EL-H0085c), IL-6 (E-EL-H6156), IL-10 (E-EL-H6156) in the cells were measured. The OD value was then determined using the Microplate Reader from Multiskan Spectrum (Thermo Scientific, Waltham, MA, USA).

### Flow cytometry

Flow cytometry was used to detect the surface markers of THP-1 cells that had been stimulated for 24 hours and collected from 6-well plates. The suspension, which had 1 × 10^6^ M1-polarized and M2-polarized cells, was then divided into 1.5 ml EP tubes and incubated with the following antibodies (PE anti-human CD86 and APC anti-human CD206; both from BioLegend, San Diego, CA, USA) on ice for 30 min in the dark. Flow cytometry (BD Biosciences, San Diego, CA, USA) was used to detect cells that had been suspended in 500 μl PBS with 3% FBS after being washed twice.

### Apoptosis detection by annexin V/PI double staining

The Annexin V-FITC Apoptosis Detection Kit (560931, BD, United States) was utilized to measure the rate of cell apoptosis, as per the manufacturer’s instructions. To summarize, the AC16 cells were seeded in 6-well plates (5 × 10^5^/well), harvested and resuspended in the binding buffer, followed by co-incubation with 5 μl of Annexin V-FITC and 5 μl of PI for 20 minutes in the dark at room temperature. Flow cytometry (BD Biosciences, San Diego, CA, USA) was utilized to analyze the cells within 1 h, and FlowJo 10.4.2 software (BD Biosciences) was then utilized to analyze the data.

### Bioinformatics analysis

At the National Center for Biotechnology Information (NCBI), GEO datasets were searched for the Sepsis-related transcriptome dataset (GSE171546). Based on the mRNA gene expression profile of model mice in time sequence, the time-related differential mRNA of CLP mice was explored. Using the R language’s ImpulseDE2 software package, the transcriptome data in the heart tissue of CLP mice was discovered. The expression matrix was analyzed by intergroup analysis and principal component analysis (PCA). The differential gene expression between CLP and control groups was predicted by a linear model analysis using the Bioconductor package “DESeq2” [18]. Using the Bioconductor package “clusterProfiler”, the key pathway information was obtained and visualized using R language [19], for Gene Ontology (GO) and Kyoto Encyclopedia of Genes and Genomes (KEGG) metabolic pathway enrichment analyses. Additionally, the “fgsea” R package was employed to perform Gene Set Enrichment Analysis (GSEA) on the entire differential gene set, and the results were visualized.

### Data analysis

GraphPad Prism 8.0 (GraphPad Inc, La Jolla, CA, USA) was employed to conduct all statistical analyses. The continuous variables were expressed as mean ± standard deviation $\left(\bar{\text{χ}}\pm \text{s}\right)$ for the measurable data. One-way ANOVA was used for comparison between groups. A Tukey test was employed to contrast the data between two sets, while ANOVA and Mann-Whitney U were employed to contrast the data between distinct groups. All tests were deemed statistically significant when the two-tailed *P* value was less than 0.05.

### Consent to participate and publish

Prior to assessing eligibility, all participants gave written informed consent and were made aware that the data would be made public.

## RESULTS

### Hmgcs2 plays a regulatory role in myocardial injury in CLP mice

**Figure 1:**
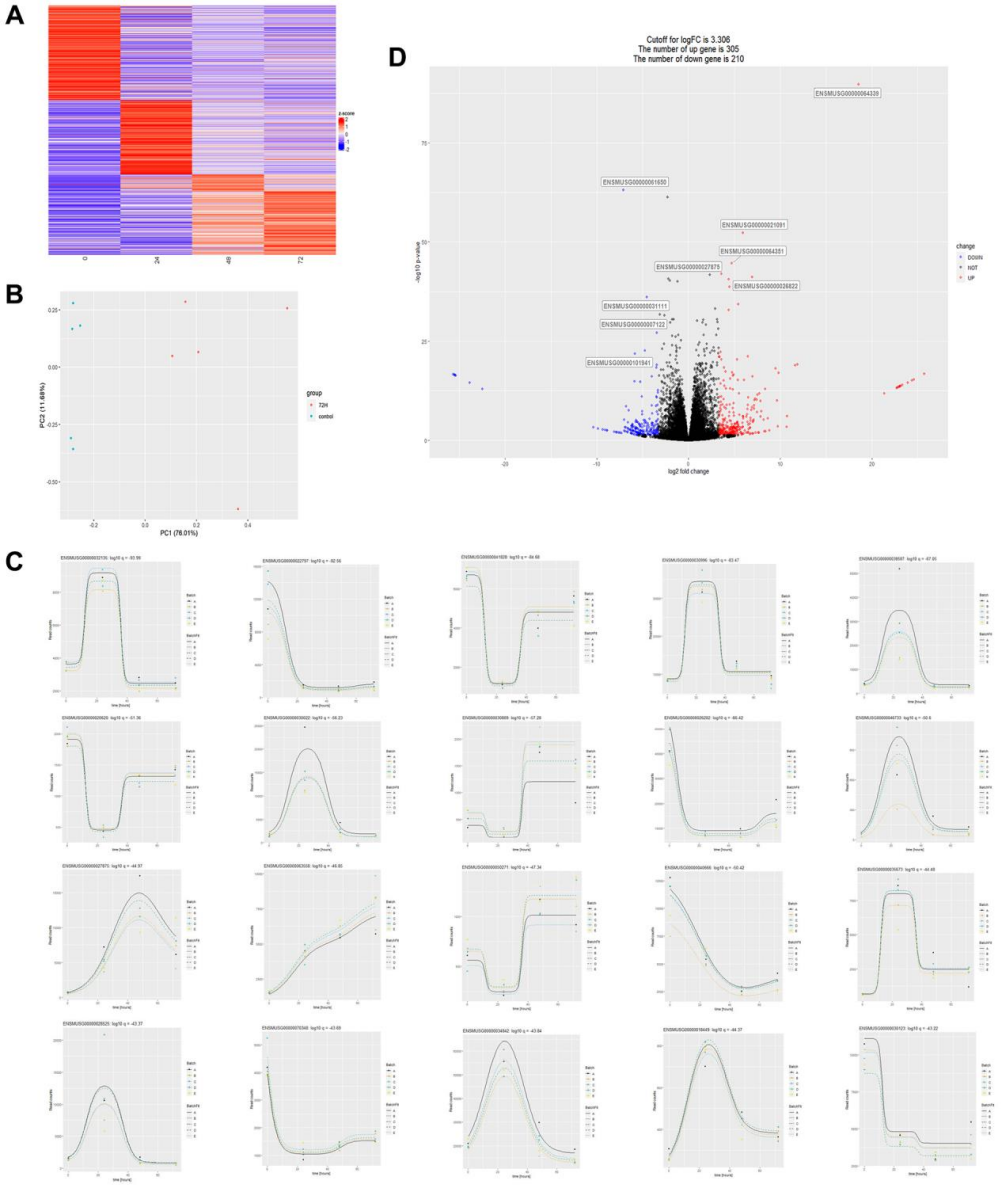
**Transcriptome data analysis of cardiac tissues from CLP mice at different times.** (**A**) Heatmap of the overall change of a gene over time, where the abscissa represents time, the vertical axis represents the amount of relative change of a gene, and the redder the color represents the more up-regulated fold of a gene, the more downregulated fold. (**B**) Principal component analysis plot (PCA) of two sets of sample data at 72 and 0 hours after CLP in the dataset. (**C**) The statistical plot of the top 20 differential genes over time, with the abscissa representing time and the ordinate representing the amount of expression of a single gene. (**D**) Differential gene volcano plot between 72H and control: 305 differentially expressed genes were up-regulated, and 210 differentially expressed genes were down-regulated with a logFC (relative expression of genes) cut-off value of 3.306, *P* < 0.05.

Transcriptome data of heart tissues from CLP mice at different times were obtained by mining GEO public data. Employing time series-based differential gene analysis, it revealed significant and diametrically opposite changes in the high and low expression of transcribed genes in CLP mice over time (Figure 1A). We performed a summary of the top 20 genes that changed significantly over time (Figure 1C). Next, we performed a differential analysis of transcribed genes 72 h after CLP. First, we performed principal component analysis on data samples from the 72-hour group (72 h) and the 0-hour control group (control) and showed that the two groups differed greatly (Figure 1B). Results of the differential analysis showed that compared to 305 genes regulated in the control group, there were 210 genes regulated in the 72 h group (Figure 1D). Alignment of the differential genes in the 72 h and control groups of CLP mice with the genes in the top 20 of temporal regulation revealed that only the gene (ENSMUSG00000027875, Hmgcs2) was significantly differentially expressed in both analysis methods, identifying it as the key differential gene.

### Inhibition of Hmgcs2 aggravates myocardial injury

**Figure 2:**
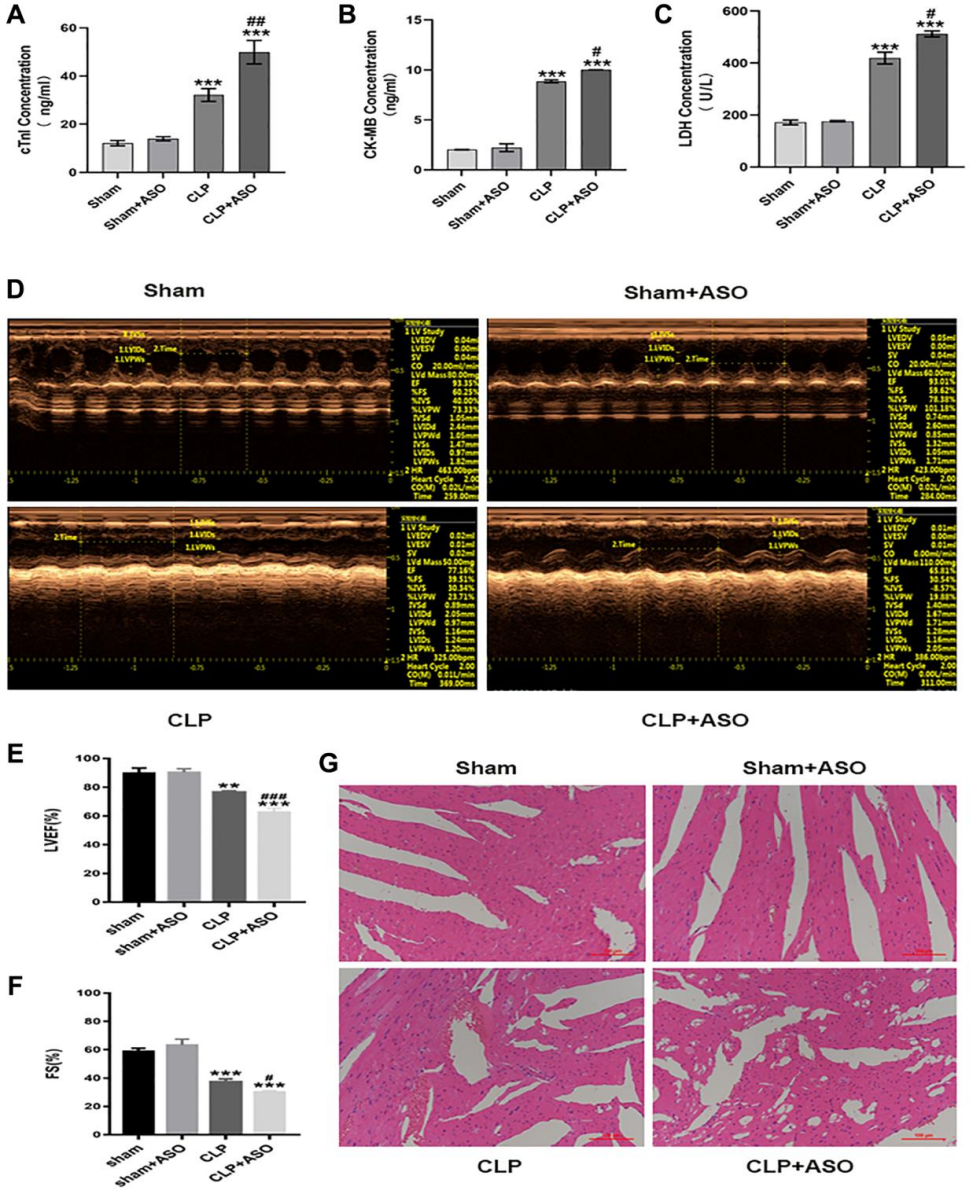
**Inhibition of hmgcs2 aggravates myocardial injury in CLP mice.** (**A**–**C**) Protein contents of cTnI, CK-MB, and LDH in the serum from mice in each group. (**D**) Echocardiography of mice in each group. (**E**, **F**) Echocardiographic statistical analysis of left ventricular ejection fraction (LVEF) and systolic fraction (FS). (**G**) H&E staining of mice heart tissues in each group (magnification 400×). Sham: sham group (*n* = 6); Sham + ASO: sham surgery plus ASO administration group (*n* = 6); CLP: septic myocarditis model group in CLP operated mice (*n* = 6); CLP + ASO: CLP plus ASO administration group (*n* = 6). ^*^P < 0.05, ^**^P < 0.01, ^***^P < 0.001: vs. sham group; ^#^*P* < 0.05, ^##^*P* < 0.01, ^###^*P* < 0.001: vs. CLP group.

A comparison of ELISA results between the sham-operated group (sham), sham plus ASO (for silencing hmgcs2) administrated group (sham + ASO), CLP model group (CLP), and CLP plus ASO administrated group (CLP + ASO) was conducted in order to further explore the regulatory role of Hmgcs2 in CLP mice. Results indicated a significant increase in the protein content of cardiac function markers LDH, CK-MB, and cTnI in the CLP Group. The serum concentrations of these proteins were augmented by ASO administration (Figure 2A–2C).

Echocardiography (Figure 2D) revealed a significant decrease in left ventricular ejection fraction (LVEF) and LV systolic function (Fractional shortening; FS) in CLP mouse hearts, as assessed by the cardiac function of each group. (Figure 2E, 2F). Moreover, the CLP + ASO group further decreased LVEF and FS.

The main pathological changes in the hearts of CLP mice included marked edema of the cardiomyocytes with vacuoles, cell deformation, necrosis, sloughing, and diffuse inflammatory cell infiltration, consistent with myocardial injury. The cardiac morphology by H&E staining (Figure 2G), comparison of the sham and CLP groups revealed that the former had caused harm to the myocardium, with myofiber tearing, cell deformation, necrosis, and inflammatory cell infiltration. The CLP + ASO group’s morphology further exacerbated the myocardial injury, while the sham + ASO group showed no difference.

### Inhibition of Hmgcs2 aggravates inflammatory response and reduces M2 macrophages in the heart

**Figure 3:**
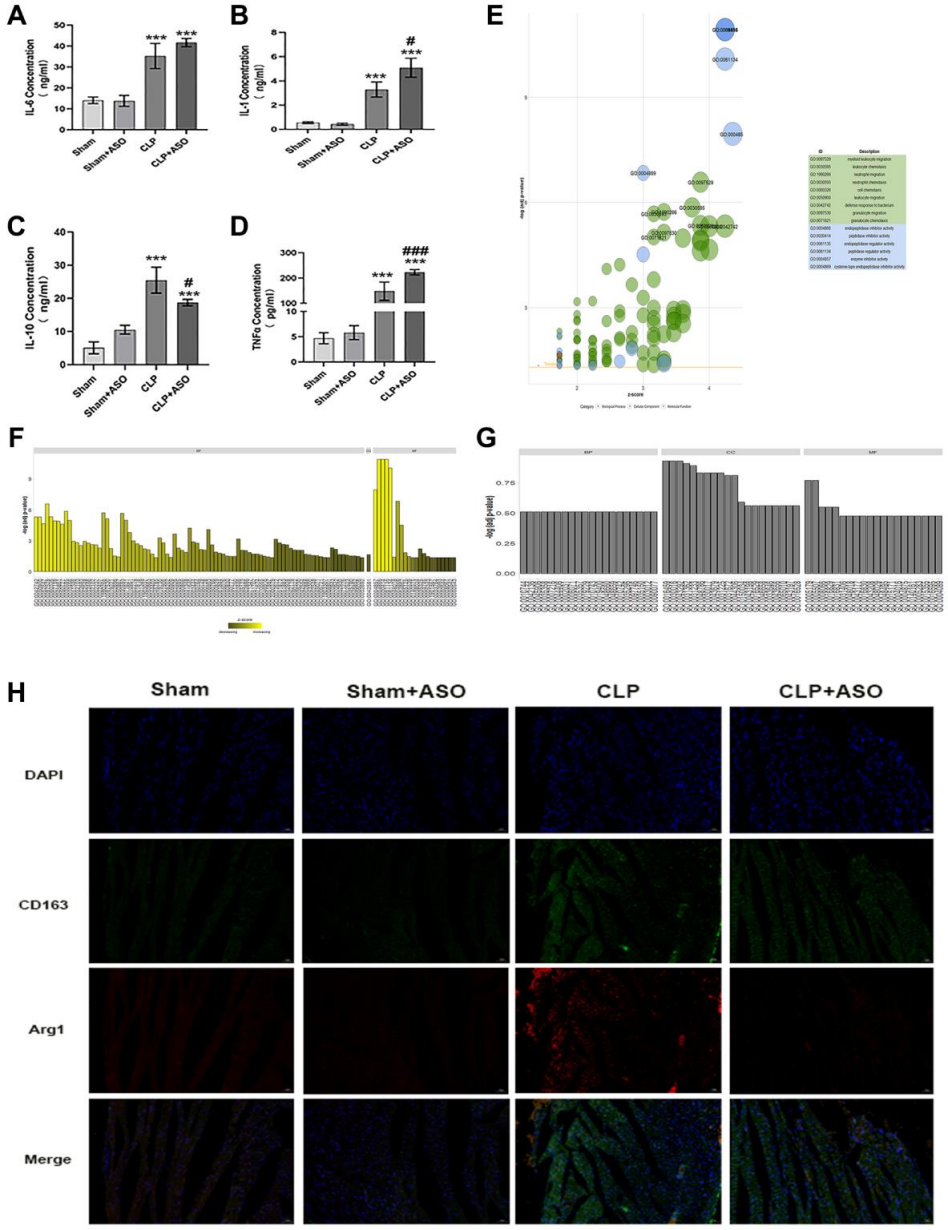
**Inhibition of Hmgcs2 aggravates inflammatory response and reduces M2 macrophages in the heart of CLP mice.** (**A**) The concentrations of the inflammatory factor IL-1β in the serum of mice in each group (ng/ml). (**B**) The concentration of IL-6 in the serum of mice in each group (ng/ml). (**C**) The concentration of IL-10 in the serum of mice in each group (ng/ml). (**D**) The concentrations of TNF-α in the serum of mice in each group (pg/ml). (**E**) GO enrichment results are exhibited in a bubble plot, with bubble size representing how many enriched genes are and different colors representing different kinds of GO units. (**F**) The bar graph exhibits the enrichment results for the up-regulated genes GO enrichment analysis results. (**G**) GO enrichment analysis results of down-regulated genes. (**H**) Immuno-fluorescence co-localization of heart samples from CLP mice. Where green fluorescence represents CD163, red fluorescence represents Arg1, blue represents nuclei (DAPI), and the bottom is a composite (merge) diagram (magnification 400×). Sham: sham group (*n* = 6); Sham + ASO: sham surgery plus ASO administration group (*n* = 6); CLP: septic myocarditis model group in CLP operated mice (*n* = 6); CLP + ASO: CLP plus ASO administration group (*n* = 6). ^*^P < 0.05, ^**^P < 0.01, ^***^P < 0.001: vs. sham group; ^#^*P* < 0.05, ^##^*P* < 0.01, ^###^*P* < 0.001: vs. CLP group.

Classically M1 and alternative M2 polarization, with pro- and anti-inflammatory properties, respectively, divide macrophages - heterogeneous immune cells with pleiotropic functions – fundamentally [12]. Characterized by proinflammatory and antimicrobial effects, M1 macrophages release copious amounts of proinflammatory factors such as IL-6, IL-1β, IFN-γ, and TNF-α, which can aggravate the inflammatory response; however, M2 macrophages, which secrete IL-10 and IL-4, have anti-inflammatory effects [20]. Our research revealed that the CLP group had notably higher levels of measured inflammatory factors. The inflammatory release of IL-1β, IL-6, and TNF-α was exacerbated by the serum concentrations of IL-1β, IL-6, IL-10, and TNF-α (Figure 3A, 3B, 3D), and ASO administration further exacerbated this. Notably, the expression of the anti-inflammatory factor IL-10 was decreased (Figure 3C), which is normally released by innate immune cells (e.g., M2 type macrophages), in the CLP + ASO group compared to the CLP group.

The top differential genes (Database of Essential Genes; DEG) were enriched through Gene Ontology (GO), with *P* < 0.05 as the filtering criterion. GO units were divided into three categories: biological process (BP), molecular function (MF), and cellular component (CC). The data indicated that DEGs were mainly enriched in GO units such as myeloid leukocyte migration, leukocyte chemotaxis, and positive regulation of macrophage-derived foam cell differentiation (Figure 3E–3G). The results demonstrated that macrophages were important for the regulation of myocardial injury in CLP mice.

Combined with the previously noted decrease in IL-10 (Figure 3C), we reasoned that Hmgcs2 might somehow be associated with M2-type macrophages in cardiac tissues of CLP mice. We mapped M2-type macrophage infiltration in the heart of CLP mice by co-immunofluorescence of CD163 and Arg1, and there was more M2-type macrophage infiltration in the CLP group compared with the sham group, which was reduced by ASO administration (Figure 3H).

### Activation of the Src/PI3K/AKT pathway by Hmgcs2 induces macrophage polarization towards M2

**Figure 4:**
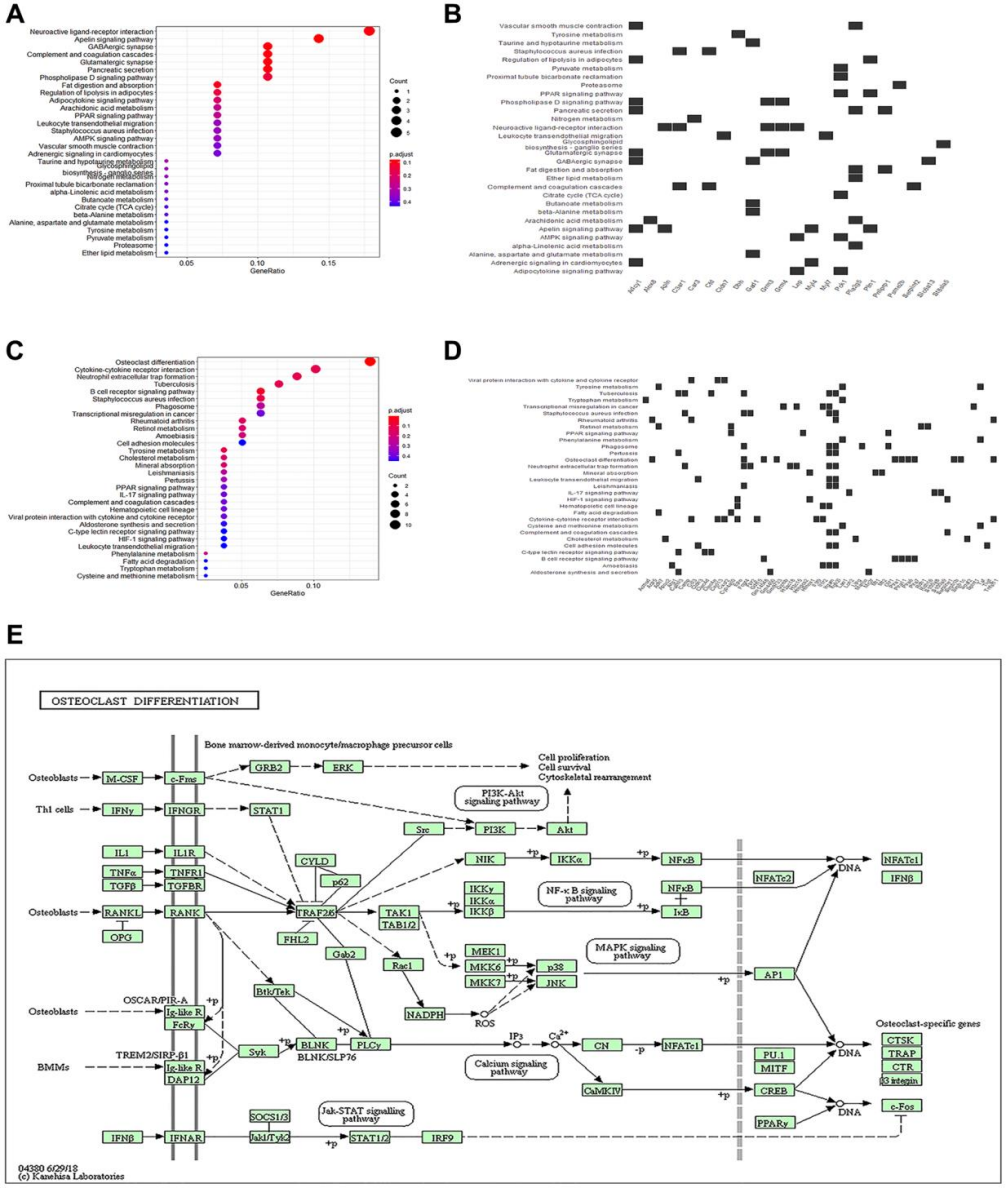
**KEGG enrichment analysis of differential genes.** (**A**) The bubble plot exhibits the KEGG enrichment analysis results of the up-regulated genes. (**B**) Heatmap of correlation between KEGG pathways and enriched genes of up-regulated genes. (**C**) The bubble plot exhibits the KEGG enrichment analysis results of down-regulated genes. (**D**) Heatmap of correlation between KEGG pathways and enriched genes of down-regulated genes. (**E**) KEGG pathway plot.

To gain insight into the regulatory mechanism of myocardial injury in CLP mice, we performed a KEGG enrichment analysis on the DEGs previously analyzed. Enriched pathways of osteoclast differentiation, B cell receptor signaling, neutrophil extracellular trap formation, cytokine receptor interaction, staphylococcus aureus infection, tuberculosis, tyrosine metabolism, rheumatoid arthritis, cholesterol metabolism, retinol metabolism, and mineral absorption were revealed in the DEGs’ up-regulated genes (Figure 4A). Downregulated genes were enriched in pathways involved in Apelin signaling, GABAergic synapses, complement and coagulation cascades, neuroactive ligand-receptor interactions, and glutamatergic synapses (Figure 4C). Enriched genes were also introduced into enriched pathways (Figure 4B, 4D). Among these, the KEGG pathways with the strongest association with macrophage polarization were osteoclast differentiation pathways (Figure 4D). Osteoclast differentiation pathways (Figure 4E) were the most strongly correlated with macrophage polarization, and activation of the Src/PI3K/Akt pathway was essential in restraining pro-inflammatory reactions and encouraging anti-inflammatory reactions in macrophages [21].

We conducted *in vitro* cell experiments to delve deeper into the regulatory part Hmgcs2 plays on macrophages. THP-1 cells were induced into macrophages by transfection or administration in four different groups, including control (NC), empty plasmid transfection (NC+ empty), Hmgcs2 over-expression (Hmgcs2), and Hmgcs2 over-expression based on the administration of the Src inhibitor PP1(Hmgcs2+ PP1).

**Figure 5:**
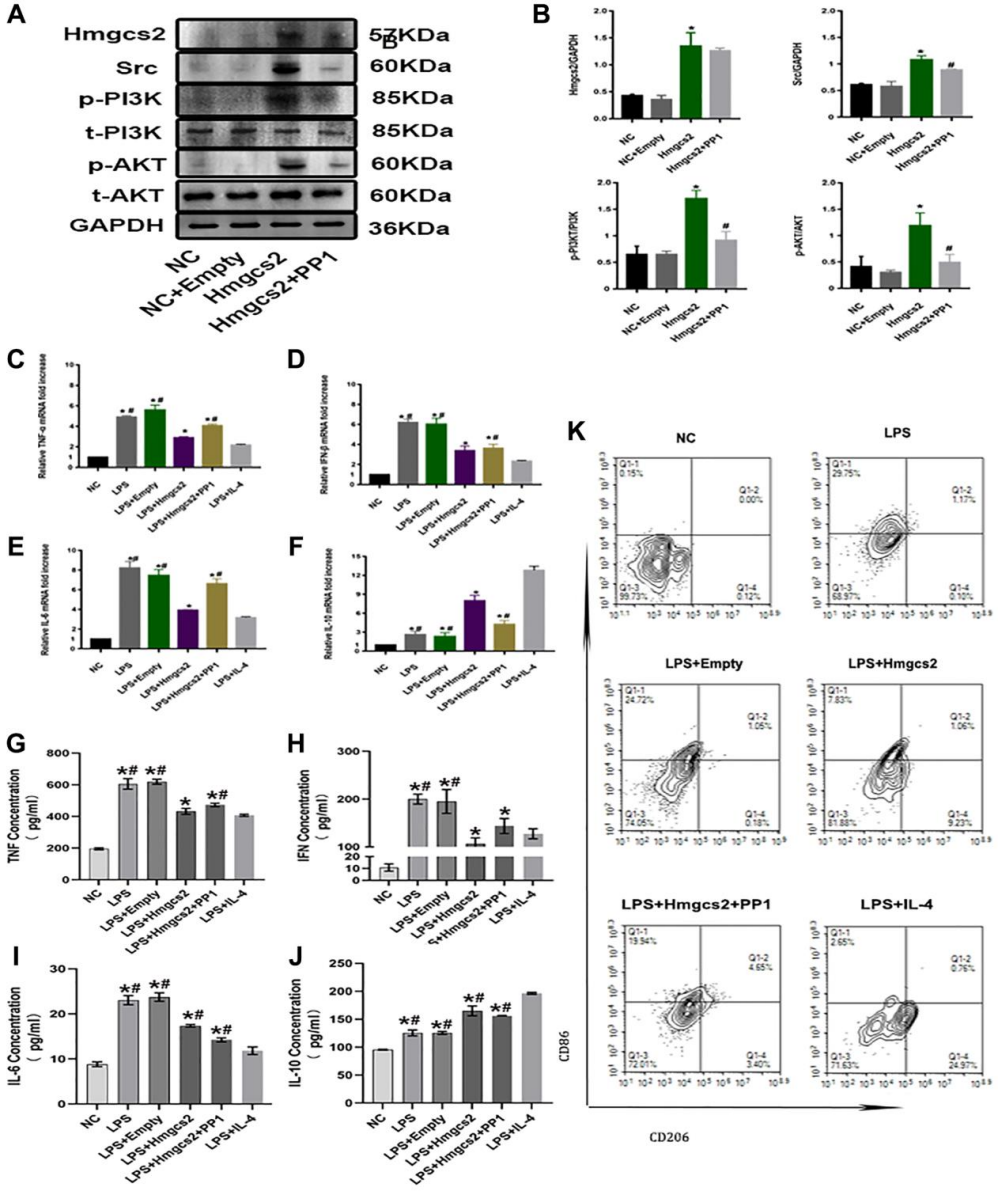
**Hmgcs2 promotes macrophage M2 polarization by activating Src/PI3K/AKT pathway.** (**A**) Relative expression of Hmgcs2 and Src/PI3K/AKT pathway-related proteins in cells from each group. (**B**) Statistical results of relative expression of Hmgcs2 and Src/PI3K/Akt pathway-related proteins of cells in each group. (**C**) The mRNA relative expression of TNF-α of macrophages in each group. (**D**) The mRNA relative expression of IFN-β of macrophages in each group. (**E**) The mRNA relative expression of IL-6 of macrophages in each group. (**F**) The mRNA relative expression of IL-10 of macrophages in each group. (**G**) Concentrations of TNF-α for macrophages in each group (pg/ml). (**H**) The concentration of IFN-β in the macrophages of each group (pg/ml). (**I**) The concentration of IL-6 in macrophages from each group (pg/ml). (**J**) The concentration of IL-10 in macrophages from each group (pg/ml). (**K**) The flow cytometry of the macrophage polarization in distinct groups. Abbreviations: NC: control group; LPS: LPS induced M1 type macrophages; LPS+ empty: M1 macrophage group transfected with empty plasmid; LPS+Hmgcs2: Hmgcs2 over-expressing M1 macrophage group; LPS+Hmgcs2+PP1: M1 macrophages with over-expression of Hmgcs2 based on PP1 administration; LPS+IL-4: LPS+IL-4 induced M2 type macrophages. ^*^P < 0.05, ^**^P < 0.01, ^***^P < 0.001: vs. NC group; ^#^*P* < 0.05, ^##^*P* < 0.01, ^###^*P* < 0.001: vs. LPS + HMGCS2 group.

First, regulation of Src/PI3K/AKT by Hmgcs2 in macrophages was demonstrated via transfection of plasmids and PP1 administration. The Hmgcs2 group displayed a rise in protein expression of Src, and a rise in phosphorylation levels of PI3K and AKT when compared to the NC group. As expected, PP1 administration decreased Src expression and decreased phosphorylation levels for PI3K and AKT in the Hmgcs2+ PP1 group, but had no effect on Hmgcs2 (Figure 5A, 5B).

Next, the inflammatory release profile of macrophages was investigated. Transcriptionally, LPS-induced mRNA expression of these inflammatory factors, including TNF-α, IFN-β, IL-6, and IL-10, was elevated in macrophages compared with the NC group (Figure 5C–5F). The ELISA experiments revealed that LPS-induced macrophages had augmented concentrations of TNF-α, IFN-β, IL-6, and IL-10 compared to the NC group, despite Hmgcs2’s over-expression causing a decrease in mRNA expression of these inflammatory factors in macrophages and an increase in transcriptional activity of IL-10. PP1 administration, however, reversed these effects. Hmgcs2’s over-expression, however, had a deleterious effect on LPS-induced TNF-α, IFN-β, and IL-6 levels, while concurrently raising IL-10 concentrations in macrophages. Again, however, PP1 administration reversed the effect of Hmgcs2 (Figure 5G–5J).

We explored the influence of Hmgcs2 on macrophage polarization, utilizing flow cytometry to quantify macrophage polarization in distinct groups. The results showed that overexpression of Hmgcs2 promoted macrophage M1 to M2 polarization, whereas PP1 administration reversed this phenomenon (Figure 5K).

### Hmgcs2 restores cardiomyocyte viability and reduces cardiomyocyte apoptosis by promoting macrophage M2 polarization

**Figure 6:**
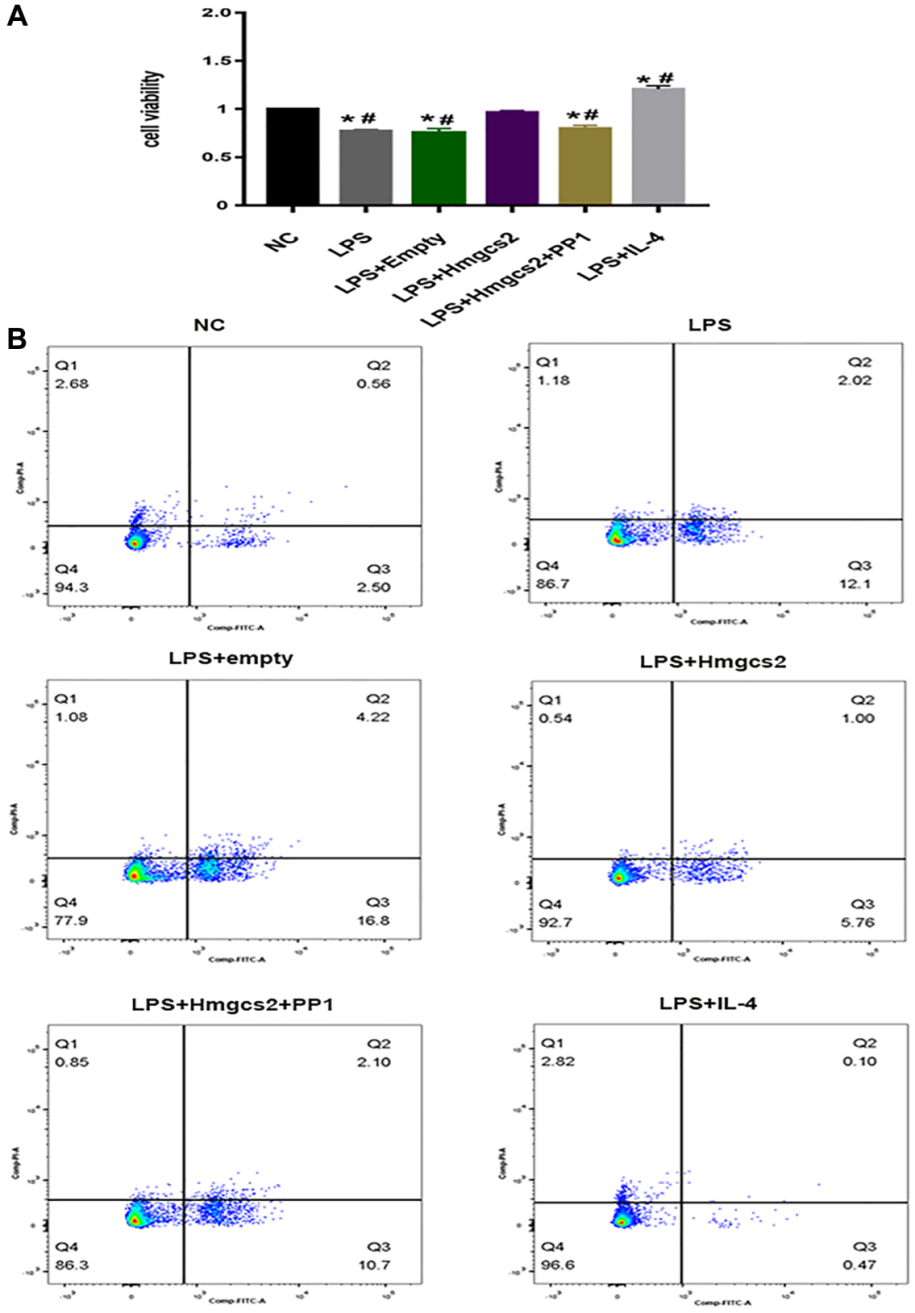
**Hmgcs2 affects cardiomyocyte activity via macrophage polarization.** (**A**) CCK-8 experiments showed the effects of different groups of macrophages on the activity of cardiomyocytes after co-culture with cardiomyocytes. (**B**) Flow cytometry experiments showed the effects of different groups of macrophages on cardiomyocyte apoptosis after co-culture with cardiomyocytes. Abbreviations: NC: control group; LPS: LPS induced M1 type macrophages; LPS+ empty: M1 macrophage group transfected with empty plasmid; LPS+Hmgcs2: Hmgcs2 over-expressing M1 macrophage group; LPS+Hmgcs2+PP1: M1 macrophages with over-expression of Hmgcs2 based on PP1 administration; LPS+IL-4: LPS+IL-4 induced M2 type macrophages. ^*^P < 0.05, ^**^P < 0.01, ^***^P < 0.001: vs. NC group; ^#^*P* < 0.05, ^##^*P* < 0.01, ^###^*P* < 0.001: vs. LPS + HMGCS2 group.

Cardiomyocyte activity was affected by Hmgcs2 by regulating macrophages in a cell co-culture experiment. CCK-8 cell viability results showed that in comparison to the NC group, LPS-induced macrophages reduced the activity of cardiomyocytes after being co-cultured with cardiomyocytes (Figure 6A), whereas cardiomyocyte activity was restored in the LPS+Hmgcs2 group co-cultured with cardiomyocytes. The LPS+IL-4 group saw a heightened level of activity in cocultured cardiomyocytes.

Furthermore, apoptosis of cardiomyocytes was investigated by flow cytometry with a co-cultured experiment. Apoptotic flow cytometry revealed that, in comparison to the NC group, cardiomyocytes in LPS-induced macrophages co-cultured with cardiomyocytes experienced an increase in apoptosis (Figure 6B); however, the LPS+Hmgcs2 group experienced a decrease in apoptosis. The LPS+IL-4 group showed the least apoptosis in co-cultured cardiomyocytes.

## DISCUSSION

The long-standing mortality rate linked to sepsis, a systemic, detrimental inflammatory reaction to infection or injury [22], has been largely attributed to multi-organ dysfunction and failure [23, 24]. Notably, cardiac dysfunction is especially common during sepsis, which is a major contributor to the high mortality rate associated with it [25–27].

Invoking multiple mechanisms linked to septic myocardial dysfunction, such as oxidative stress overabundance, cardiomyocyte apoptosis, cardiac contractile malfunction, and persistent inflammation [28–30], the treatment of this potentially fatal condition is often inadequate and only marginally successful, particularly when the present comprehension of its pathophysiology is still disturbingly unclear. We conducted a public data mining analysis of the transcriptome results of CLP mice to explore the regulatory mechanism of sepsis-induced myocardial injury, discovering that Hmgcs2 was highly expressed in the cardiac tissues of the mice and could be a crucial factor in the condition (Figure 1).

Hmgcs2 is one of the main control points of ketogenesis during ketone body metabolism. The potential for ketone bodies to have beneficial effects on the heart, especially in the case of experimental ischemia/reperfusion injury, could be a cardioprotective factor, potentially due to an augmented concentration of cardiac mitochondria or an increase in the amount of essential oxidative phosphorylation mediators [7, 8]. Hmgcs2 generates ketone bodies including acetoacetate, acetate, and β- Hydroxybutyric acid (β OHB) [31]. Additionally, β OHB was found to be capable of inhibiting NF-κB through partial translocation, as well as reducing the expression of pro-inflammatory proteins (iNOS, COX-2) or cytokines (TNF-α, IL-1β, IL-6, CCL2/MCP-1) [32], it was also observed that β OHB could inhibit the NLRP3 inflammasome in bone marrow-derived macrophages. The present study demonstrated that Hmgcs2 had a positive effect on myocardial function in CLP mice, and its inhibition further exacerbated the symptoms (Figure 2), quite likely caused by aggravating inflammation (Figure 3A–3D). Our research revealed a remarkable rise in the levels of inflammatory factors in the CLP group, such as serum concentrations of IL-1β, IL-6, IL-10, and TNF-α. Furthermore, ASO administration further exacerbated the inflammatory release of IL-1β, IL-6, and TNF-α (Figure 3A–3C). Notably, the expression of the anti-inflammatory factor IL-10 was decreased (Figure 3D). Interleukin-10 (IL-10), a cytokine, is a major anti-inflammatory agent and plays a pivotal part in controlling and terminating inflammatory reactions [33]. The up-regulating of the production of IL-10 has beneficial effects in sepsis mouse models [34–36]. Macrophages, especially M2 macrophages, are the most prominent cell population expressing the immunoregulatory cytokine IL-10 [37]. Studies have demonstrated that IL-10 secreted by macrophages, particularly immune cells, is a critical factor in the battle against sepsis.

Macrophages, heterogeneous immune cells with pleiotropic functions [38], are fundamentally divided into classically M1 and alternative M2 polarization with pro- and anti-inflammatory property, respectively [39]. The production of inflammatory mediators by macrophages in sepsis hyper-inflammation is noteworthy, as they are the main factor in M1 polarization [40]. Consequently, the introduction or administration of anti-inflammatory M2 macrophages during sepsis hyper-inflammation is advantageous. The production of M1-like macrophages, stemming from the iNOS pathway, which yields citrulline and NO from arginine, and M2-like macrophages, from the arginase pathway, which yields ornithine and urea from arginine [15], is the source of the M1/M2 polarity, which is caused by two opposing pathways. CD86 and CD80 are the known M1 markers, and CD163 and CD206 are the known M2 markers [17]. Characterized by proinflammatory and antimicrobial effects, M1 macrophages release a great deal of proinflammatory factors such as IL-6, IL-18, IFN-γ, and TNF-α, which can exacerbate the inflammatory response; however, M2 macrophages are distinguished by their anti-inflammatory properties [20, 41]. GO enrichment analysis showed the association of macrophages with myocardial injury in CLP mice (Figure 1). The inhibition of Hmgcs2 exacerbated the inflammatory response and decreased M2 macrophages in the heart (Figures 2 and 3), that is Hmgcs2 can regulate M2 polarization of macrophages to repair myocardial injury induced by sepsis.

The mechanism by which Hmgcs2 regulated macrophage polarization was further investigated, first by identifying the critical role of Src/PI3K/Akt through KEGG enrichment analysis (Figure 4). The PI3K/Akt signaling pathway is known to be a crucial factor in macrophage M2 activation in reaction to surfactant protein A or IL-4. Additionally, IL-10, TGF-β, and BMP-7 are known to induce M2 polarization [21]. However, Hmgcs2 could bind with peroxisome proliferator-activated receptors α (PPAR α) directly to promote Src activity [42]. Concordant with prior studies, the present study found that overexpressed Hmgcs2 could activate the Src/PI3K/Akt pathway to promote macrophage M2 polarization and thus reduce the release of pro-inflammatory factors from macrophages (Figure 5). Finally, to demonstrate the direct effect of macrophage polarization on cardiomyocytes, M2-type macrophages were able to reduce cardiomyocyte apoptosis and enhance cardiomyocyte activity when co-cultured with them *in vitro* (Figure 6).

Studies have indicated a correlation between a lack of ketogenesis and a range of inflammatory ailments, with Hmgcs2 being the essential rate-limiting enzyme for ketogenesis. Ketogenic insufficiency by ASO- mediated Hmgcs2 knockdown in adult mice leads to liver inflammation and injury [43, 44]. Hmgcs2 ketogenesis acts as an endogenous protective program during acute pancreatitis by suppressing inflammatory macrophage activation [45]. In our study, an endogenous protective mechanism of Hmgcs2 during septic myocardial injury was proposed. Namely, Hmgcs2 acts as a protective program by suppressing pro-inflammatory macrophage activation and promoting anti-inflammatory phenotype, in which the phenotypic shift of macrophages is achieved through Hmgcs2 regulated Src/PI3K/AKT. Providing a novel theoretical basis for prevention and treatment, it offers a protective mechanism against septic complications.

## CONCLUSION

To conclude, the current research revealed the protective power of Hmgcs2 against septic myocardial injury, by stimulating macrophage M2 polarization to diminish inflammation induced by sepsis and decreasing cardiomyocyte apoptosis to lessen myocardial injury through the activation of the Src/PI3K/Akt pathway (Supplementary Figure 1).

## Supplementary Materials

Supplementary Figure 1
