# Supplementary material for: Hmgcs2 regulates M2 polarization of macrophages to repair myocardial injury induced by sepsis
Source: Aging (Albany NY). 2023 Aug 9;15(15):7794–810. doi: 10.18632/aging.204944 (PMC10457052; doi:10.18632/aging.204944)
Supplement: Supplementary Figure 1 [file aging-15-204944-s001.pdf]

## SUPPLEMENTARY FIGURE

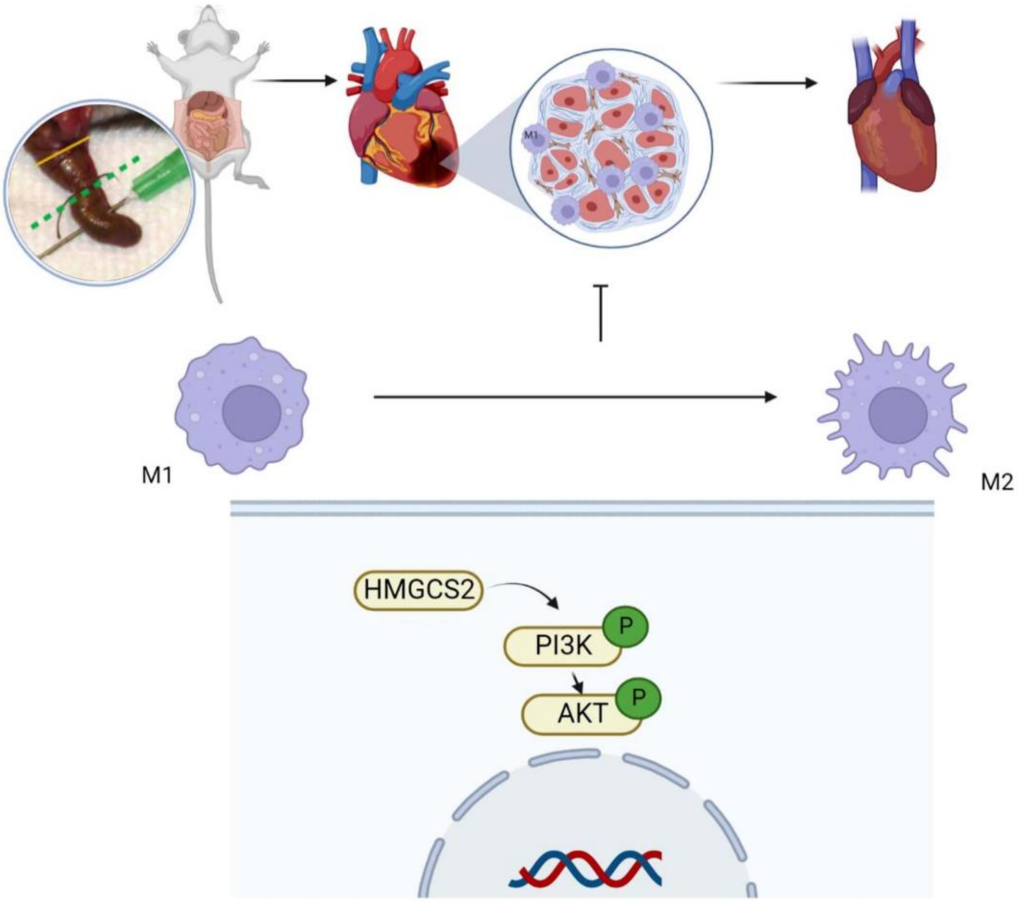


**Supplementary Figure 1. The mechanisms of action of Hmgcs2 as an endogenous protective program in septic myocardial injury.**
